# Dynamics of malaria transmission and susceptibility to clinical malaria episodes following treatment of *Plasmodium falciparum* asymptomatic carriers: results of a cluster-randomized study of community-wide screening and treatment, and a parallel entomology study

**DOI:** 10.1186/1471-2334-13-535

**Published:** 2013-11-12

**Authors:** Alfred B Tiono, Moussa W Guelbeogo, N Falé Sagnon, Issa Nébié, Sodiomon B Sirima, Amitava Mukhopadhyay, Kamal Hamed

**Affiliations:** 1 Centre National de Recherche et de Formation sur le Paludisme (CNRFP), 01 BP 2208, Ouagadougou 01, Burkina Faso; 2 Novartis Healthcare Private Limited, Hyderabad, India; 3 Novartis Pharmaceuticals Corporation, One Health Plaza, East Hanover, NJ 07936-1080, USA

**Keywords:** Malaria, *Plasmodium falciparum*, Asymptomatic carriers, Community screening, Entomological inoculation rate, Parasite density, Disease transmission

## Abstract

**Background:**

In malaria-endemic countries, large proportions of individuals infected with *Plasmodium falciparum* are asymptomatic and constitute a reservoir of parasites for infection of newly hatched mosquitoes.

**Methods:**

Two studies were run in parallel in Burkina Faso to evaluate the impact of systematic identification and treatment of asymptomatic carriers of *P. falciparum*, detected by rapid diagnostic test, on disease transmission and susceptibility to clinical malaria episodes. A clinical study assessed the incidence of symptomatic malaria episodes with a parasite density >5,000/μL after three screening and treatment campaigns ~1 month apart before the rainy season; and an entomological study determined the effect of these campaigns on malaria transmission as measured by entomological inoculation rate.

**Results:**

The intervention arm had lower prevalence of asymptomatic carriers of asexual parasites and lower prevalence of gametocyte carriers during campaigns 2 and 3 as compared to the control arm. During the entire follow-up period, out of 13,767 at-risk subjects, 2,516 subjects (intervention arm 1,332; control arm 1,184) had symptomatic malaria. Kaplan-Meier analysis of the incidence of first symptomatic malaria episode with a parasite density >5,000/μL showed that, in the total population, the two treatment arms were similar until Week 11–12 after campaign 3, corresponding with the beginning of the malaria transmission season, after which the probability of being free of symptomatic malaria was lower in the intervention arm (logrank p < 0.0001). Similar trends were observed in infants and children <5 years and in individuals ≥5 years of age. In infants and children <5 years old who experienced symptomatic malaria episodes, the geometric mean *P. falciparum* density was lower in the intervention arm than the control arm. This trend was not seen in those individuals aged ≥5 years. Over the year, monthly variation in mosquito density and entomological inoculation rate was comparable in both arms, with September peaks in both indices.

**Conclusion:**

Community screening and targeted treatment of asymptomatic carriers of *P. falciparum* had no effect on the dynamics of malaria transmission, but seemed to be associated with an increase in the treated community’s susceptibility to symptomatic malaria episodes after the screening campaigns had finished. These results highlight the importance of further exploratory studies to better understand the dynamics of disease transmission in the context of malaria elimination.

## Background

In 2010, as many as 1.2 million individuals are estimated to have died from malaria, revealing the considerable burden that this disease continues to place on many endemic areas in sub-Saharan Africa [1]. However, in contrast to this trend, a number of countries in this region have experienced a decline in malaria transmission over the past decade due to the implementation of sustained malaria control and prevention strategies [2-6]. Following these promising results, stakeholders within the malaria community have begun to consider malaria elimination in certain epidemiological settings [7,8]. Appropriate strategies for drug use in an elimination context have consequently been a subject of discussion, with mass drug administration (MDA) and systematic screening for parasite carriage followed by treatment being reviewed [9,10].

MDA involves the administration of antimalarials to entire populations without knowledge of who is infected. In countries with high malaria endemicity, the majority of MDA programs have failed in their attempts to interrupt transmission or provided only a transient success [11,12]. Given the lack of evidence for sustained success, the fear that widespread use of antimalarials in these programs could speed up the development of parasite resistance and the potential high economic cost of such interventions, MDA is not currently recommended by the World Health Organization [13].

Screening and treatment only of infected individuals is an alternative to MDA. In malaria-endemic countries, a large proportion of *Plasmodium falciparum* infections are asymptomatic [14-16]. As a consequence, the systematic identification and treatment of asymptomatic carriers could potentially decrease disease transmission by reducing the pool of parasites carried by these individuals [17,18]. A recent simulation suggests that mass screening and treatment could be a cost-effective intervention in areas with medium-to-high levels of transmission that have achieved moderate coverage with insecticide-treated nets [19].

The description of the epidemiology of malaria is often focused on clinical parameters such as prevalence of parasitemia. However, entomological information such as vector species composition and density, proportion of infected mosquitoes, entomological inoculation rate (EIR), and insecticide resistance status are key transmission parameters essential to the planning of control measures. This article reports results from two studies run in parallel: a clinical study to evaluate the impact of systematic treatment of *P. falciparum* asymptomatic carriers at a community level on the incidence of symptomatic malaria episodes with a parasite density >5,000/μL; and an entomological study to determine the effect of the screening and treatment intervention on malaria transmission as measured by EIR. The studies took place in the health district of Saponé, about 50 km south of Ouagadougou in Burkina Faso, which is an area with marked seasonal *P. falciparum* malaria transmission from June to November [20]. The null hypothesis in this study was that there would be no difference in terms of EIR between the intervention (mass screening and treatment of *P. falciparum* asymptomatic carriers) and control arms.

## Methods

### Clinical study methods

The full study methodology was published by Tiono *et al*. [21]. A brief summary of the methodology is given here.

This was a single-center, controlled, parallel, cluster-randomized study that evaluated the effect of systematic treatment of *P. falciparum* asymptomatic carriers on the incidence of symptomatic malaria episodes in children (<5 years) and adults over a 12-month period after completion of three community screening and treatment campaigns, compared with no treatment of asymptomatic carriers. In total, 18 clusters (each comprising one village) were randomized and assigned in a 1:1 ratio to the intervention or control arm.

Before the study implementation phase, all inhabitants of the 18 clusters were provided with long-lasting insecticide-treated nets (LLINs; OLYSET® nets [Sumitomo Chemical Co, Ltd, Tokyo, Japan]). Compliance with mosquito net use was checked every two months during home visits to the trial participants by Demographic Surveillance System (DSS) fieldworkers.

**Figure 1:**
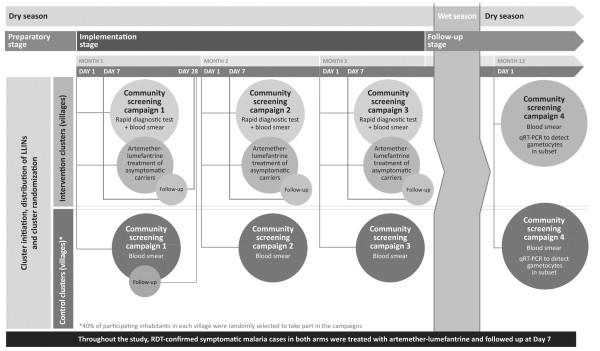
**Single-center, controlled, parallel, cluster-randomized, 12-month prospective study.** Reproduced from Tiono *et al*. 2013 [21].

During the implementation phase, inhabitants of the intervention and control clusters participated in the three screening campaigns that took place ~1 month apart between January and April 2011, before the start of the rainy season. A fourth campaign was conducted in January 2012 after the rainy season had ended to mark the end of the study at ~12 months (Figure 1). At each campaign, finger-prick blood samples were taken from the entire study population in the intervention arm and a randomly selected 40% in the control arm for microscopic screening for *P. falciparum* asexual forms and gametocytes. In the intervention arm, the population was also screened using a rapid diagnostic test (RDT; First Response® Malaria Ag, Premier Medical Corp Ltd., Nani-Daman, India) to identify asymptomatic carriers. Those individuals with a positive RDT received treatment with artemether-lumefantrine (20 mg artemether and 120 mg lumefantrine [Coartem®, Novartis Pharma AG, Basel, Switzerland]) or artemether-lumefantrine dispersible, twice a day for three consecutive days. Subjects in the control arm were not screened by RDT – microscopy alone with delayed reading was used to ensure that study personnel and screened subjects remained unaware of subject status.

Following the third screening campaign, study participants in both arms were followed up for passive detection of symptomatic malaria throughout the wet season, when malaria transmission is high. Participants were encouraged to report to their local healthcare facility or clinic as soon as they felt unwell. An RDT was performed for all participants attending the local healthcare facility with confirmed fever (axillary temperature ≥37.5°C) or history of fever within the last 24 hours. On day 1 of campaign 4, a blood sample was also collected for gametocyte assessment by quantitative reverse transcription-polymerase chain reaction (qRT-PCR), which was conducted in 1,999 randomly selected subjects drawn from the entire intervention group and a 40% subgroup of the control population. In this study, a case of symptomatic malaria was defined as fever or history of fever within the previous 24 hours and a *P. falciparum* asexual parasite count >5,000/μL. Each episode of symptomatic malaria was treated and the patient followed up at day 7. Parasitological cure was assessed by microscopy for each symptomatic malaria episode on day 7.

The blood films obtained during visits and for symptomatic malaria assessment were air-dried and Giemsa-stained for examination by a light microscope fitted with a 100 X oil immersion lens at a single laboratory. At least 200 thick film fields were examined before a slide was declared negative. When *P. falciparum* was present, a count of the asexual forms against leukocytes was made using a tally counter. Counting was done based on at least 200 leukocytes according to WHO standards. If less than 10 parasites were identified from the 200 leukocyte screen, counting was extended to 1,000 leukocytes. If *P. falciparum* gametocytes were seen, a gametocyte count was performed against 1,000 leukocytes. All slides were read by two independent microscopists. If the ratio of densities from the first two readings was >1.5 or <0.67, or if less than 30 parasites were counted with an absolute difference of more than 10 in the number of parasites, the slide was evaluated by a third microscopist. The definitive result was the mean of the parasite density of the two most concordant reading results.

Microscopist competency was evaluated through two equivalent quality control (EQC) programs. The first EQC was carried out by College of American Pathology proficiency testing and included a set of 5 slides provided to each microscopist for reading thrice a year. The second EQC was performed by WHO (National Institute for Communicable Diseases) and involved the reading of a set of 20 slides every quarter by each microscopist. Only those with a score of at least 80%, graded as ‘competent’ , were involved in the reading of trial participants’ slides.

This trial is registered with ClinicalTrials.gov, number NCT01256658.

#### Ethics section

The protocol and the proposed informed consent form were reviewed and approved by the Centre National de Recherche et de Formation sur le Paludisme Institutional Review Board and by the National Ethical Committee for Health Research of Burkina Faso. Prior to study initiation, a community meeting was held in each of the selected clusters to discuss the study with the community. The freedom of each individual household and household member to decide on participation was discussed to minimize the potential influence of key opinion leaders in each cluster. Individual informed consent was obtained from each participant during a visit to the household before any study procedure.

### Entomology study methods

#### Mosquito collection and processing

From the 18 clusters selected for the clinical study, 10 were randomly selected for the entomology study. To achieve random selection, two lists containing the nine villages from each arm were generated. Using the random function in Excel (Microsoft Corporation, Redmond, WA), each village on the intervention list and the control list was assigned a number between 1 and 9. The villages in each arm were then listed in ascending order on the basis of the randomly assigned number. The first five villages from each list were selected for inclusion in the entomology study.

Beginning at the initiation of the first screening campaign and continuing weekly until screening campaign 4 (i.e. ~12 months later), mosquitoes were collected from 10 randomly selected houses in each of the 10 selected clusters. To randomly select the houses for mosquito collection, a list was generated from the DSS database that contained the names of the heads of each household in each cluster. The random number function in Excel was then used to select 10 individual households from the list for each of the 10 clusters.

Mosquitoes were collected using the indoor pyrethrum spray catch method [22]. These collections were conducted on one day each week between the hours of 07:00 and 10:00 AM to obtain those mosquitoes that had fed on the inhabitants of the house during the previous night.

#### Determination of mosquito density, sporozoite index, human biting rate and entomological inoculation rate (EIR)

All mosquitoes collected were transported to the laboratory where the abdomens of the females were examined under a dissecting microscope to classify them into their respective blood feeding stages: unfed, blood-fed, semi-gravid, and gravid.

From each collection round, the head-thorax portion of 200 randomly selected blood-fed, semi-gravid and gravid female *Anopheles gambiae* s.l. was dissected from the abdomen. The random selection was done by using the random number function in Excel to select mosquitoes on the basis of the unique ID numbers given to each insect. The head-thorax portions were tested by an enzyme-linked immunosorbent assay (ELISA) that specifically detects *P. falciparum* circumsporozoite protein (CSP) antigen to allow estimation of the *P. falciparum* sporozoite index according to the method described by Burkot *et al*. (1984) and modified by Wirtz *et al.* (1987) [23,24]. To determine the number of mosquitoes that had fed on humans, an ELISA was performed on the abdomen portions to screen for IgG in the blood meals [25].

#### Identification of mosquito species

All mosquitoes collected were morphologically identified. In addition, molecular analysis was used to confirm the morphological classification of *An. gambiae* s.l. for at least 100 insects from each collection. These were randomly selected by ID number as previously described. Mosquitoes positive for the ELISA *P. falciparum* CSP also underwent molecular classification. Mosquitoes belonging to *An. gambiae* s.l. were identified to the species level using a standard PCR technique as described by Fanello *et al.*[26].

#### Insecticide target site mutation (kdr) detection

A subsample of 500 *An. gambiae* s.l. mosquitoes (50 from each intervention and control cluster) were randomly selected from each collection (as described above) to undergo testing by PCR according to Martinez-Torres *et al.* (1998) and Ranson *et al.* (2000) for detection of the *kdr* gene mutation that confers resistance to pyrethroids [27,28]. To obtain an accurate representation of the *kdr* allele frequency in each village of the study area, all *An. gambiae* s.l. collected were eligible for *kdr* screening regardless of whether they were alive or dead.

### Statistical methods

This study was analyzed at the cluster level, i.e. the cluster was considered as an experimental unit and observations were based on individual subjects in each cluster. All hypothesis testing, except human biting rate and EIR, was conducted one-sided at a 0.05 level of significance. No adjustments for multiplicity were performed.

To account for both the occurrence and timing of the first RDT-confirmed symptomatic malaria episode with a parasite density >5,000/μL (SMRC_5000_), survival analysis was carried out using PROC LIFETEST in SAS®, assuming hazard ratio remains constant over time, and considering only the number of subjects at risk at that time point of consideration as denominator. Time was measured in days from the day of diagnosis of asymptomatic carriage to first SMRC_5000_ in the study period. The survival probability was estimated based on a Kaplan-Meier estimate. Missing time was censored at time of withdrawal or completion of the study period including immigrant and emigrant subjects.

Mosquito density was expressed as the number of female mosquitoes collected per household. The human biting rate was determined by dividing the number of mosquitoes that had fed on humans by the number of people who had slept in the house during the night before collection. From these data, the daily EIR was the product of the human biting rate (number of bites/person/night) and the proportion of sporozoite-positive mosquitoes. The daily EIR was multiplied by the number of days in the corresponding month to obtain an estimate of the monthly EIR. Then, the monthly EIRs in each arm were summed up to obtain the cumulative annual EIR. To examine the influence of the intervention on human biting rates and EIR, a two-sided Wilcoxon rank sum test at 95% level of significance was adopted.

## Results

### Clinical study demographics and baseline characteristics

**Figure 2:**
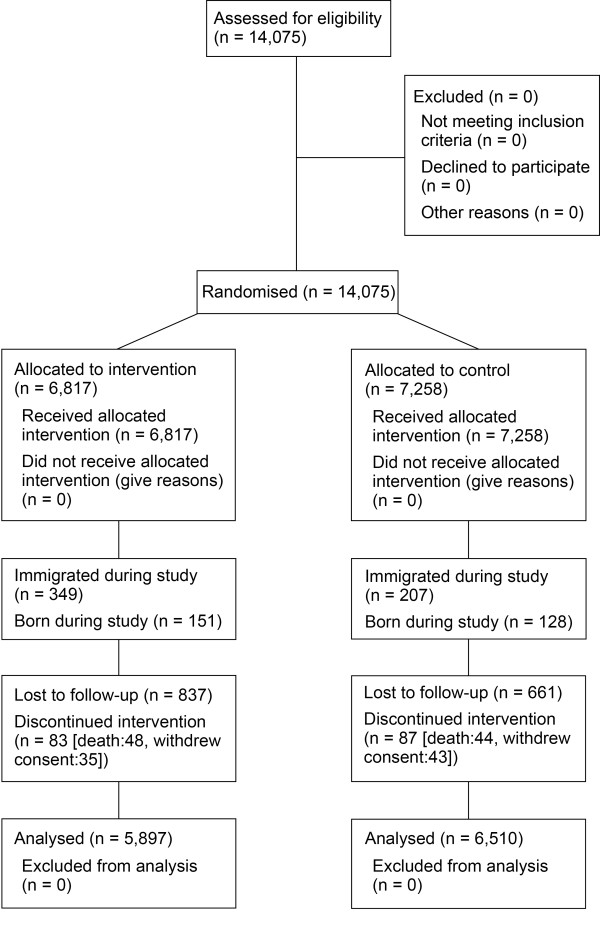
**Consort chart.** Reproduced from Tiono *et al*. 2013 [21].

These results were reported previously by Tiono *et al.* (2013) [21]. A total of 6,817 persons in the intervention arm and 7,258 persons in the control arm were recruited and enrolled in the clinical study (Figure 2). The intervention and control arms were similar in terms of demographic characteristics with the exception of ethnicity; the intervention arm had a higher proportion of Fulani (8.7% vs 3.8%). Overall, 96.1% of the population in the intervention arm who consented to participation were tested by RDT and treated accordingly. The primary outcomes of this study are reported in Tiono *et al*. (2013) [21].

### Prevalence of asymptomatic carriers

The prevalence of asymptomatic carriage of asexual parasites over time is presented in Table 1. The mean percentage of carriers in the intervention arm was much lower at campaign 2 and campaign 3 than in the control arm, but showed only a small difference at campaign 4.

**Table 1:** Prevalence of microscopy-confirmed asymptomatic carriers of asexual parasites over time by study arm

**Study arm**	**Campaign 1/Day 1**	**Campaign 2/Day 1**	**Campaign 3/Day 1**	**Campaign 4/Day 1**
	**Mean (SD)**			
Intervention	42.8 (5.67)	4.1 (1.62)	2.8 (0.92)	34.4 (3.92)
Control	47.5 (8.05)	35.7 (4.94)	32.2 (9.26)	37.8 (6.37)

### Incidence of first symptomatic malaria episodes in all ages

**Figure 3:**
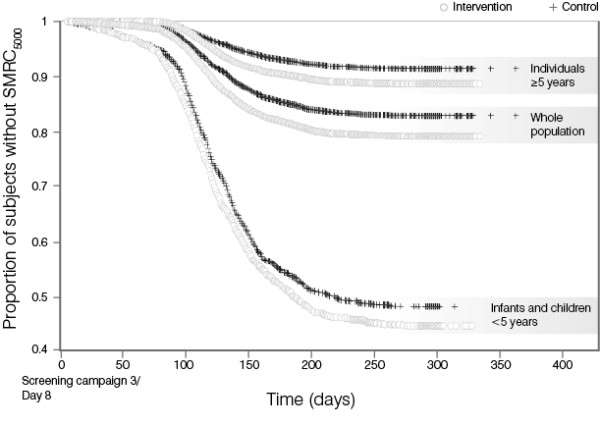
**Kaplan-Meier plot of incidence of first SMRC**_**5000 **_**following screening campaign 3 – cluster level data.**

Kaplan-Meier analysis of the incidence of first SMRC_5000_ following screening campaign 3 showed that in the total population the two treatment arms were similar until Week 11–12, i.e. the beginning of the malaria transmission season. After this time, the probability of being free of malaria was lower in the intervention arm (logrank p <0.0001). Similar trends were observed in infants and children <5 years (logrank p = 0.0356) and in individuals ≥5 years of age (logrank p <0.0001; Figure 3, Additional file 1). Subjects who discontinued before day 8 of screening campaign 3 were excluded from these analyses. Age was considered on day 1 of screening campaign 1.

**Figure 4:**
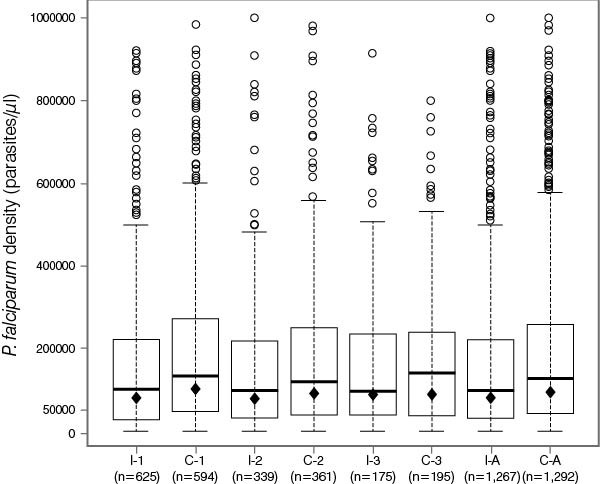
**Distribution of *P. falciparum *density during SMRC**_**5000 **_**in infants and children <5 years of age following screening campaign 3.** I = intervention arm; C = control arm; numbers 1–3 indicate first, second and third episodes, and A any episode; n = number of subjects at each episode; central rectangles span the first quartile to the third quartile (IQR); the line inside each rectangle shows the median; the whiskers that extend from each box indicate the range of values that are outside of the intra-quartile range but close enough not to be considered outliers (a distance less than or equal to 1.5*IQR); ♦ = geometric mean.

**Figure 5:**
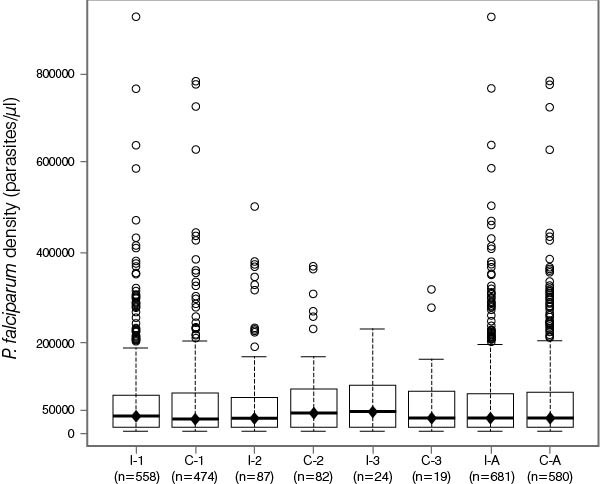
**Distribution of *P. falciparum *density during SMRC**_**5000 **_**in individuals ≥5 years of age following screening campaign 3.** I = intervention arm; C = control arm; numbers 1–3 indicate first, second and third episodes, and A any episode; n = number of subjects at each episode; central rectangles span the first quartile to the third quartile (IQR); the line inside each rectangle shows the median; the whiskers that extend from each box indicate the range of values that are outside of the intra-quartile range but close enough not to be considered outliers (a distance less than or equal to 1.5*IQR); ♦ = geometric mean.

Figures 4 and 5 illustrate the distribution of *P. falciparum* density during the different SMRC_5000_ episodes in infants and children <5 years and in individuals aged ≥5 years of age. In infants and children <5 years of age who experienced symptomatic malaria episodes, the geometric mean of *P. falciparum* density was lower in the intervention arm than the control arm. The parasite density (parasites/μL) in the intervention vs the control arm was 82,203 vs 104,120 during the first episode, 81,583 vs 95,248 during the second episode, 88,599 vs 91,514 during the third episode, and 83,532 vs 97,966 at any episode (Figure 4). This trend was not seen in those individuals aged ≥5 years. The parasite density in the intervention vs the control arm was 34,176 vs 33,614 during the first episode, 40,109 vs 37,499 during the second episode, 47,225 vs 35,915 during the third episode, and 35,783 vs 34,182 at any episode (Figure 5).

The proportion of individuals in the total population carrying gametocytes during any clinical malaria episode of any parasite density was 3.0% (95% CI 2.5% – 3.7%) and 3.8% (95% CI 3.2% – 4.6%) respectively in the intervention and the control arms. Similar patterns were observed between arms when the participants were stratified by age category.

**Figure 6:**
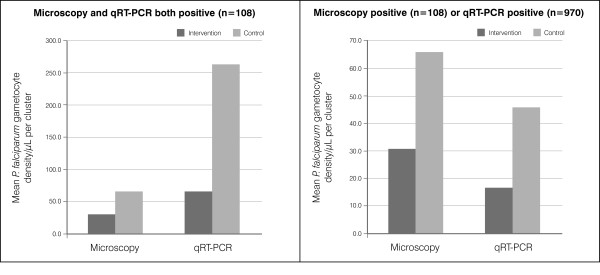
**Mean *P. falciparum *** **gametocyte density at screening campaign 4.**

Gametocyte density as measured by microscopy at the end of the trial was at least 2-fold higher in the control arm than in the intervention arm. When gametocyte density was measured by qRT-PCR, the difference between the two arms was even greater (Figure 6).

### Entomology study results

**Figure 7:**
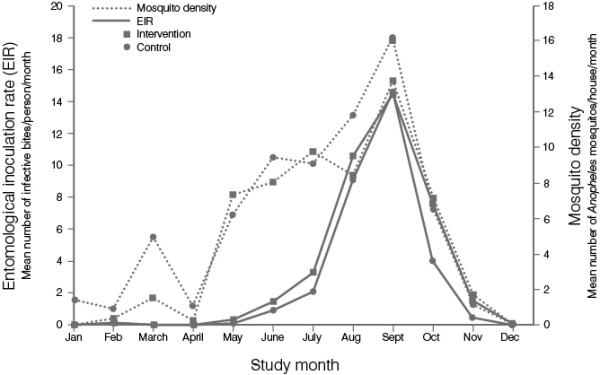
**Temporal variation of *Anopheles *** **density and entomological inoculation rate (EIR) by study arm.**

From January to December 2011, a total of 12,466 mosquitoes were collected across the 10 clusters (6,677 in the control arm and 5,789 in the intervention arm). The species composition collected in the two arms of the study was similar (Table 2). The frequency of the *kdr* gene mutation was slightly higher in the control arm (0.71; SD 0.16) than the intervention arm (0.65; SD 0.12). The intervention arm mean (SD) biting rate was 1.12 (1.20) bites per person per night, vs 1.12 (1.19) bites per person per night in the control arm. Over the year, EIR and mosquito density results were also comparable in both arms, with peaks for both observed in September (Table 3, Figure 7). Although the monthly EIR appeared to be slightly higher in the intervention arm than the control arm throughout the study period (except September, when it was slightly lower in intervention arm), the difference was not statistically significant (p = 0.7086, considering all 12 months). Similar results were also obtained considering only the months July to December (p = 0.8182).

**Table 2:** Entomological results by study arm

	**Intervention**	**Control**
Mosquito species		
*Anopheles gambiae* s.l.	91.7%	92.7%
*Anopheles funestus*	0.1%	0.2%
Other *Anopheles*	0.6%	0.4%
*Culex*	7.4%	6.5%
*Aedes*	0.2%	0.2%
*Anopheles gambiae* s.l. composition		
*Anopheles arabiensis*	13%	14%
*Anopheles coluzzi*	61%	57%
*Anopheles gambiae*	26%	28%
Frequency of *kdr* West African mutation (SD)	0.65 (0.12)	0.71 (0.16)
Mean number of mosquitoes/house (SD)	6.30 (5.89)	5.96 (2.67)
Mean number of bites/person/night (SD)	1.12 (1.20)	1.12 (1.19)
EIR (i.e. cumulative number of infective bites/person/year)	39.46	31.42

**Table 3:** Monthly variation of entomological indices in study arms

	**Number of fed mosquitoes collected**		**Human blood index**		**Mean human biting rate (number of bites/person/night)**		**Sporozoite index**	
**Month**	**Intervention**	**Control**	**Intervention**	**Control**	**Intervention**	**Control**	**Intervention**	**Control**
January	1	15	0.00	1.00	0.00	0.24	0.00	0.00
February	29	48	0.83	0.85	0.08	0.15	0.06	0.03
March	35	144	1.00	0.92	0.22	0.75	0.00	0.00
April	9	37	1.00	1.00	0.04	0.32	0.00	0.00
May	261	189	1.00	1.00	1.58	1.03	0.01	0.00
June	492	634	0.97	0.86	1.89	1.65	0.03	0.02
July	610	630	0.93	0.75	2.07	1.77	0.05	0.04
August	712	808	0.96	0.96	1.84	1.89	0.19	0.16
September	993	1238	0.98	0.90	3.83	4.23	0.13	0.12
October	502	413	0.99	0.92	1.46	1.25	0.17	0.10
November	122	78	0.93	0.80	0.41	0.19	0.13	0.08
December	3	3	1.00	1.00	0.02	0.04	0.16	0.00

## Discussion

Community screening and targeted treatment of asymptomatic carriers of *P. falciparum* malaria was associated with a negative impact on the incidence of first symptomatic malaria episode with a parasite density >5,000/μL. The probability of developing a first episode of symptomatic malaria was higher in the intervention arm than in the control arm; however, the cumulative incidence of malaria was similar in both arms (Tiono *et al.* 2013) [21].

The results may suggest that clearance of asymptomatic infections increases an individual’s risk of contracting a symptomatic malaria episode. A similar ‘rebound’ effect has been observed following the use of chemoprophylaxis in malaria-endemic areas [29-31]. It has been proposed that asymptomatic carriage might be a form of tolerance to *P. falciparum* infection that protects against the development of clinical episodes [32]. Consequently, clearance of these infections might have rendered individuals more susceptible to reinfection and development of clinical malaria. A number of reports have also indicated that while semi-immunity to malaria can be acquired in highly endemic areas by the age of 5 years, this immunity wanes rapidly without ongoing parasite exposure [33,34]. As shown in Figure 3, children <5 years of age, individuals of at least 5 years of age and the total population were all at risk of developing a symptomatic malaria episode with a parasite density >5,000/μL after the three screening campaigns. These findings seem to confirm that in the absence of continual exposure, any immunity an individual has acquired to clinical disease is relatively short-lived.

The development of clinical and parasitological immunity to malaria is marked by the ability of individuals to control disease and parasite density [34]. In this study, children in the intervention arm <5 years of age tended to develop clinical signs and symptoms at lower parasite densities than their peers in the control arm; a situation that was reversed in older children and adults. While it is difficult to draw conclusions from these findings, it is possible that, overall, the treatment of asymptomatic carriers had some effect on the development of immunity that may differ with the age of the subject. In younger children in the intervention arm, this effect on immunity seemed to make them more susceptible to symptomatic disease (or more likely to notice its signs and symptoms) at lower parasite densities. However, it may also be that the lower parasite density reflects only an earlier diagnosis of disease due to the fact that the caregivers of young children in the intervention arm were more prompt to report to their healthcare facility upon the first signs of illness. In older children and adults, the treatment appears to affect the ability to control parasite density, suggesting possible interference with the anti-parasite immunity which is known to restrict the density of asexual parasitemia.

Among other factors, malaria transmission depends on the presence of infectious sexual parasites (gametocytes) in human peripheral blood. While individuals with subpatent gametocytemia are able to infect mosquitoes, previous research carried out in Burkina Faso has shown that individuals with microscopically detected gametocytes are two-fold more likely to be infectious compared to those with submicroscopic gametocytes (68.2% vs 31.7%; p = 0.001) [35]. Although gametocyte density in our study was far greater in the control arm compared to the intervention arm, transmission indices were similar in both arms. This suggests that there was no difference in transmission ecology between the study arms, and that transmission potential of individuals in the intervention arm was not affected by the screening and treatment. Moreover, although not supported by these results, an enhanced post-treatment transmission potential in the absence of a change in transmission dynamic in the intervention arm cannot be excluded. It is known that the presence of immunity to *P. falciparum* sexual stages is an essential factor for reducing human-to-mosquito transmission of malaria parasites by preventing the fertilization and development of the parasite in the mosquito midgut. In addition, it has been shown in previous work that naturally acquired immunity against Pfs48/45 and Pfs230 (antigens specific to the sexual stages of *P. falciparum*) is a function of recent exposure rather than of cumulative exposure to gametocytes [36]. Therefore, with the known gametocidal effect of artemether-lumefantrine on immature gametocytes and the significant reduction of gametocyte prevalence in the intervention vs the control arm following the screening campaigns, it is possible that immunity against Pfs48/45 and Pfs230 could be impaired, thus increasing the infectivity of treated individuals to the mosquitoes.

A potential confounding factor is the invasion of intervention clusters by infected mosquitoes from surrounding villages that did not receive LLINs and thereby contributed to the spread of infection; however, as such invasion is also likely to occur in the control clusters the effect of this factor on the overall results is likely to be marginal. Additionally, RDT sensitivity (vs microscopy) during the three screening campaigns was 92.4%, 84.0% and 77.8%. These findings suggest that by using RDT and subsequent microscopic confirmation a notable proportion of asymptomatic carriers were undetected. The use of more sensitive molecular techniques, possibly in combination with RDT, may merit exploration in future mass screening and treatment interventions.

Overall, the malaria transmission dynamic in the study arms is comparable to previous reports from the area [37]. The human biting rates observed in our study at the September peak (3.83 in the intervention arm and 4.23 in the control arm) were far lower than the 26.7 bites/person/night that was previously reported in the area [36]. This is probably due to the high coverage of the study population with LLINs and the reduction in mosquito aggressiveness following mass bednet use [38,39]. The prevalence of *kdr* mutation observed was comparable to that observed in 2010 by Badolo *et al.* in a village located 30 km from Ouagadougou with the same agricultural practices as the study area [40].

## Conclusion

Community screening and targeted treatment of asymptomatic carriers of *P. falciparum* seemed to be associated with an increase in the treated community’s susceptibility to malaria episodes once the screening campaigns had finished. In the context of malaria elimination, these results clearly show the importance of further exploratory studies to better understand the dynamics of disease transmission and possible immunological factors involved. They also underline the fact that clearing the reservoir of malaria parasites with effective antimalarial drugs might not be enough to interrupt malaria transmission in high-transmission settings; association with malaria transmission-blocking tools might be required. In the absence of an effective malaria transmission-blocking vaccine, drugs with known transmission-blocking potential such as primaquine [41] and ivermectin [42] might have a significant role to play in this regard, and this should be explored.

## Competing interests

AT has received honoraria from Novartis Pharma AG, Basel, Switzerland to attend Advisory Board meetings to discuss this study and manuscript. AM is an employee of Novartis Healthcare Private Limited, and KH is an employee of Novartis Pharmaceuticals Corporation. MG, NS, IN and SS declared no competing interests.

## Authors’ contributions

All authors were involved in the design of the study, data interpretation, and defining the content for and critically reviewing the manuscript. AT, MG, NS, IN and SS were involved in data collection, while AT, MG, AM and KH conducted the data analysis. AT, MG, NS, AM and KH were involved in writing the manuscript. All of the authors had full access to data in the study, discussed the results, reviewed the draft manuscript and agreed on the final version. KH, the corresponding author, had final responsibility for the decision to submit the manuscript for publication. Editorial assistance was provided by Louisa Reed from PreScript Communications, with funding from Novartis Pharma AG.

## Pre-publication history

The pre-publication history for this paper can be accessed here:

http://www.biomedcentral.com/1471-2334/13/535/prepub

## Supplementary Material

Additional file 1 Incidence of first symptomatic malaria episode with a parasite density >5,000/μL. Click here for file
