# Supplementary material for: Dynamics of malaria transmission and susceptibility to clinical malaria episodes following treatment of Plasmodium falciparum asymptomatic carriers: results of a cluster-randomized study of community-wide screening and treatment, and a parallel entomology study
Source: BMC Infect Dis. 2013 Nov 12;13:535. doi: 10.1186/1471-2334-13-535 (PMC4225764; doi:10.1186/1471-2334-13-535)
Supplement: Additional file 1 — Incidence of first symptomatic malaria episode with a parasite density >5,000/μL. [file 1471-2334-13-535-S1.docx]

**Supplemental material: Incidence of first symptomatic malaria episode with a parasite density >5,000/µL**

n*: Total number of subjects at risk at the start of the interval
n: Cumulative number of subjects having a symptomatic malaria episode with a parasite density >5,000/µL

|  | **Whole population** | | | |
| --- | --- | --- | --- | --- |
|  | **Intervention arm** | | **Control arm** | |
| **Time Interval after campaign 3 (Week)** | **n*/n** | **Cumulative probability of not having symptomatic malaria (95% CI)** | **n*/n** | **Cumulative probability of not having symptomatic malaria (95% CI)** |
| [1, 2] | 6640/4 | 0.999 (0.999, 1.000) | 7127/11 | 0.998 (0.998, 0.999) |
| [3, 4] | 6607/21 | 0.997 (0.995, 0.998) | 7100/22 | 0.997 (0.996, 0.998) |
| [5, 6] | 6573/36 | 0.995 (0.993, 0.996) | 7066/41 | 0.994 (0.992, 0.996) |
| [7, 8] | 6528/54 | 0.992 (0.990, 0.994) | 7019/57 | 0.992 (0.990, 0.994) |
| [9, 10] | 6494/79 | 0.988 (0.985, 0.991) | 6984/85 | 0.988 (0.985, 0.991) |
| [11,12] | 6442/174 | 0.973 (0.969, 0.977) | 6931/142 | 0.980 (0.977, 0.983) |
| [13, 14] | 6318/349 | 0.946 (0.941, 0.952) | 6852/292 | 0.958 (0.954, 0.963) |
| [15, 16] | 6123/594 | 0.908 (0.901, 0.915) | 6685/484 | 0.931 (0.925, 0.937) |
| [17, 18] | 5866/821 | 0.873 (0.865, 0.881) | 6479/646 | 0.907 (0.901, 0.914) |
| [19, 20] | 5622/976 | 0.849 (0.840, 0.858) | 6305/811 | 0.884 (0.876, 0.891) |
| [21, 22] | 5419/1087 | 0.832 (0.822, 0.841) | 6124/929 | 0.867 (0.859, 0.875) |
| [23, 24] | 5243/1156 | 0.821 (0.811, 0.830) | 5966/1002 | 0.856 (0.848, 0.864) |
| [25, 26] | 5063/1212 | 0.811 (0.802, 0.821) | 5848/1049 | 0.849 (0.841, 0.857) |
| [27, 28] | 4977/1266 | 0.803 (0.793, 0.812) | 5725/1106 | 0.841 (0.832, 0.849) |
| [29, 30] | 4878/1296 | 0.798 (0.788, 0.808) | 5604/1134 | 0.836 (0.828, 0.845) |
| [31, 32] | 4820/1305 | 0.796 (0.786, 0.806) | 5551/1153 | 0.833 (0.825, 0.842) |
| [33, 34] | 4786/1317 | 0.794 (0.784, 0.804) | 5517/1169 | 0.831 (0.822, 0.840) |
| [35, 36] | 4688/1325 | 0.793 (0.783, 0.803) | 5448/1175 | 0.830 (0.821, 0.839) |
| [37, 38] | 4584/1330 | 0.792 (0.782, 0.802) | 5362/1181 | 0.829 (0.820, 0.838) |
| [39, 40] | 4536/1332 | 0.792 (0.782, 0.802) | 5295/1183 | 0.829 (0.820, 0.838) |
| [41, 42] | 4505/1332 | 0.792 (0.782, 0.802) | 5271/1184 | 0.829 (0.820, 0.838) |

|  | **Individuals ≥5 years** | | | |
| --- | --- | --- | --- | --- |
|  | **Intervention arm** | | **Control arm** | |
| **Time Interval after campaign 3 (Week)** | **n*/n** | **Cumulative probability of not having symptomatic malaria (95% CI)** | **n*/n** | **Cumulative probability of not having symptomatic malaria (95% CI)** |
| [1, 2] | 5100/0 | 1.000 (1.000, 1.000) | 5665/3 | 0.999 (0.999, 1.000) |
| [3, 4] | 5100/0 | 1.000 (1.000, 1.000) | 5653/3 | 0.999 (0.999, 1.000) |
| [5, 6] | 5100/0 | 1.000 (1.000, 1.000) | 5653/4 | 0.999 (0.999, 1.000) |
| [7, 8] | 5100/0 | 1.000 (1.000, 1.000) | 5624/8 | 0.999 (0.998, 1.000) |
| [9, 10] | 5100/1 | 1.000 (1.000, 1.000) | 5595/16 | 0.997 (0.996, 0.999) |
| [11,12] | 5037/25 | 0.995 (0.993, 0.997) | 5564/40 | 0.993 (0.991, 0.995) |
| [13, 14] | 4985/99 | 0.980 (0.976, 0.984) | 5529/98 | 0.982 (0.979, 0.986) |
| [15, 16] | 4906/212 | 0.958 (0.952, 0.963) | 5467/167 | 0.970 (0.966, 0.974) |
| [17, 18] | 4785/320 | 0.936 (0.929, 0.943) | 5394/232 | 0.958 (0.953, 0.964) |
| [19, 20] | 4668/392 | 0.922 (0.914, 0.929) | 5320/295 | 0.947 (0.941, 0.953) |
| [21, 22] | 4575/438 | 0.912 (0.904, 0.920) | 5244/338 | 0.939 (0.933, 0.945) |
| [23, 24] | 4508/470 | 0.906 (0.898, 0.914) | 5188/375 | 0.932 (0.926, 0.939) |
| [25, 26] | 4444/494 | 0.901 (0.893, 0.909) | 5126/401 | 0.928 (0.921, 0.935) |
| [27, 28] | 4402/522 | 0.895 (0.887, 0.904) | 5056/428 | 0.923 (0.916, 0.930) |
| [29, 30] | 4336/539 | 0.892 (0.883, 0.900) | 5007/445 | 0.920 (0.912, 0.927) |
| [31, 32] | 4299/544 | 0.891 (0.882, 0.899) | 4972/454 | 0.918 (0.911, 0.925) |
| [33, 34] | 4277/550 | 0.889 (0.881, 0.898) | 4955/465 | 0.916 (0.909, 0.923) |
| [35, 36] | 4230/554 | 0.888 (0.880, 0.897) | 4915/468 | 0.915 (0.908, 0.923) |
| [37, 38] | 4210/558 | 0.888 (0.879, 0.896) | 4890/472 | 0.915 (0.907, 0.922) |
| [39, 40] | 4162/558 | 0.888 (0.879, 0.896) | 4867/473 | 0.914 (0.907, 0.922) |
| [41, 42] | 4162/558 | 0.888 (0.879, 0.896) | 4845/474 | 0.914 (0.907, 0.922) |

|  | **Infants and children <5 years** | | | |
| --- | --- | --- | --- | --- |
|  | **Intervention** | | **Control** | |
| **Time Interval after campaign 3 (Week)** | **n*/n** | **Cumulative probability of not having symptomatic malaria (95% CI)** | **n*/n** | **Cumulative probability of not having symptomatic malaria (95% CI)** |
| [1, 2] | 1540/4 | 0.997 (0.995, 1.000) | 1462/8 | 0.995 (0.991, 0.998) |
| [3, 4] | 1525/21 | 0.986 (0.980, 0.992) | 1447/19 | 0.987 (0.981, 0.993) |
| [5, 6] | 1502/36 | 0.976 (0.969, 0.984) | 1433/37 | 0.975 (0.966, 0.983) |
| [7, 8] | 1477/54 | 0.964 (0.955, 0.974) | 1404/49 | 0.966 (0.957, 0.975) |
| [9, 10] | 1454/78 | 0.948 (0.937, 0.960) | 1389/69 | 0.952 (0.941, 0.963) |
| [11,12] | 1421/149 | 0.901 (0.886, 0.916) | 1367/102 | 0.929 (0.916, 0.942) |
| [13, 14] | 1333/250 | 0.832 (0.813, 0.851) | 1323/194 | 0.864 (0.846, 0.882) |
| [15, 16] | 1217/382 | 0.742 (0.719, 0.764) | 1218/317 | 0.776 (0.755, 0.798) |
| [17, 18] | 1081/501 | 0.660 (0.635, 0.684) | 1085/414 | 0.707 (0.683, 0.731) |
| [19, 20] | 954/584 | 0.601 (0.576, 0.627) | 985/516 | 0.634 (0.608, 0.659) |
| [21, 22] | 844/649 | 0.555 (0.529, 0.580) | 880/591 | 0.579 (0.553, 0.605) |
| [23, 24] | 735/686 | 0.526 (0.500, 0.552) | 778/627 | 0.552 (0.526, 0.578) |
| [25, 26] | 619/718 | 0.498 (0.472, 0.524) | 722/648 | 0.536 (0.509, 0.562) |
| [27, 28] | 575/744 | 0.476 (0.449, 0.502) | 670/678 | 0.511 (0.484, 0.537) |
| [29, 30] | 543/757 | 0.464 (0.438, 0.491) | 597/689 | 0.501 (0.475, 0.528) |
| [31, 32] | 521/761 | 0.461 (0.434, 0.487) | 580/699 | 0.493 (0.466, 0.519) |
| [33, 34] | 516/767 | 0.455 (0.428, 0.482) | 565/704 | 0.488 (0.461, 0.515) |
| [35, 36] | 461/771 | 0.451 (0.424, 0.477) | 534/707 | 0.485 (0.458, 0.512) |
| [37, 38] | 383/772 | 0.449 (0.423, 0.476) | 472/709 | 0.483 (0.456, 0.510) |
| [39, 40] | 381/774 | 0.447 (0.420, 0.474) | 430/710 | 0.482 (0.455, 0.509) |
